# Immune Metabolism in TH2 Responses: New Opportunities to Improve Allergy Treatment — Cell Type-Specific Findings (Part 2)

**DOI:** 10.1007/s11882-022-01058-7

**Published:** 2022-12-15

**Authors:** Y.-J. Lin, A. Goretzki, H. Rainer, J. Zimmermann, Stefan Schülke

**Affiliations:** Vice President’s Research Group 1: Molecular Allergology, Paul-Ehrlich-Institut, Paul-Ehrlich-Str. 51-59, 63225 Langen, Germany

**Keywords:** Allergy, Immune metabolism, Immunotherapy, Warburg, Fatty acid oxidation, Oxidative phosphorylation, APC, ILC2, Th2

## Abstract

**Purpose of Review:**

Over the last years, we have learned that the metabolic phenotype of immune cells is closely connected to the cell’s effector function. Understanding these changes will allow us to better understand allergic disease pathology and improve allergy treatment by modulating immune metabolic pathways. As part two of a two-article series, this review reports on the recent studies investigating the metabolism of the cell types involved in allergies and discusses the initial application of these discoveries in allergy treatment.

**Recent Findings:**

The cell types involved in allergic reactions display pronounced and highly specific metabolic changes (here discussed for epithelial cells, APCs, ILC2s, mast cells, eosinophils, and Th2 cells). Currently, the first drugs targeting metabolic pathways are tested for their potential to improve allergy treatment.

**Summary:**

Immune-metabolic changes observed in allergy so far are complex and depend on the investigated disease and cell type. However, our increased understanding of the underlying principles has pointed to several promising target molecules that are now being investigated to improve allergy treatment.

## Introduction

Cell metabolism is a highly complex network of biochemical reactions that aims at providing the cell with both energy and the building blocks of its cellular components. Recent breakthrough publications have demonstrated that, in immune cells, cellular metabolism is both closely connected to and regulates the respective cells’ effector function by not only providing large amounts of energy needed in activated immune cells but also supplying metabolic intermediates that are required to form important immune effector molecules [1].

Better understanding these intricate connections already has and will in future further increase our concept of disease pathology and allow for new treatment strategies. Initial findings have strongly suggested immune-metabolism to also be highly relevant in the field of allergies.

In the first part of this two-part review series, we have already covered the basics of immune-metabolism and reported on the recently published studies on disease-specific metabolic changes associated with allergies [1]. In the second part, we will summarize (I) the recently published findings in the most important cell types involved in allergic reactions and (II) discuss the initial application of these discoveries in allergy treatment.

## Cell Type-Specific Findings in Allergy

Besides investigating the contribution of different metabolic pathways in different allergic diseases (see above), many studies also focused on isolated cell types. Here, we summarize the findings from the last 3 years.

### Epithelium/Epithelial Cells

As the first line of contact with our non-sterile exterior, epithelial cells and their secreted mediators play a crucial role in activating other immune cells by producing chemokines and cytokines in response to either pathogen-associated molecular patterns or allergens [2]. Therefore, the link between their metabolism and effector function is of great interest for allergy development and treatment.

Li et al. found in 2019 that the regeneration of airway stem-like variant club cells (vclub cells) (former called “Clara cells”, which are bronchiolar exocrine cells) was dependent on autophagy [3]. In addition, autophagy was furthermore shown to enhance the differentiation of airway progenitor club cells into ciliated or goblet cells in an OVA-induced mouse lung inflammation model [3]. Additionally, the authors found that autophagy facilitates glucose uptake with glucose transporter 1 (Glut1), promoting the proliferation of Club cells and positively regulating the differentiation of vClub cells into Club cells while simultaneously inhibiting their further differentiation into ciliated or goblet cells [3]. In contrast, complete inhibition of glycolysis inhibited both the differentiation and proliferation of the progenitor cells [2]. Therefore, glucose metabolism was shown to be essential for the function, proliferation, and differentiation capacity of airway progenitor cells [3].

Nishimura and colleagues investigated the contribution of signal transducer and activator of transcription 3 (STAT3), a transcription factor that can be activated by several cytokines and is involved in apoptosis, proliferation, and differentiation of airway epithelia cells, to HDM-induced allergic airway inflammation [4•]. Based on a mouse model with airway epithelia cell-specific, doxycycline (DOX)-inducible STAT3-deficiency (STAT3-cKO), the HDM-induced airway inflammation was evaluated using flow cytometry and analyzed via qPCR and RNA-sequencing [4•]. Interestingly, in STAT3-cKO mice, house dust mite (HDM)-treatment was shown to enhance airway inflammation compared to wild type animals (STAT3-WT) (evidenced by increased total number of cells, eosinophils, CD4^+^ T cells, goblet cells, and histopathological score) [4•]. In RNA-sequencing analysis, the gene coding for the fatty acid desaturating enzyme stearoyl-coenzyme A desaturase 1 (SCD1) showed the highest upregulation in HDM-treated STAT3-WT mice compared to PBS-treated controls [4•]. Furthermore, a gene cluster relating to lipid biosynthetic processes was also upregulated [4•]. Interestingly, HDM-treated STAT3-WT and STAT3-cKO mice shared 45 differently expressed genes when HDM was combined with the intraperitoneal application of an SCD1-inhibitor [4•]. In line with the upregulation of SCD1, these genes were related to fatty acid- and lipid-biosynthesis processes, suggesting a correlation between STAT3, SCD1, and lipid metabolism [4•]. Therefore, the authors concluded that STAT3 and SCD1 are involved in the suppression of HDM-induced inflammation, making STAT3, SCD1, and overall lipid metabolism interesting targets in the prevention of allergic inflammation [4•].

### Antigen Presenting Cells

It has recently been reviewed by Sun and colleagues in 2021 that dendritic cell (DC)-mediated immune responses are also being regulated by FAO [5]. In an inactive state, DCs perform predominantly catabolic pathways, e.g., fatty acid oxidation (FAO), amino acid metabolism, Krebs cycle, and oxidative phosphorylation (OxPhos) [5]. Activated DCs, in turn, switch to increased anabolism in the form of, e.g., glycolysis, fatty acid synthesis, and production of reactive oxygen species (ROS) [5]. According to the authors, the role of FAO should be further investigated to gain better insight inside into the complex mechanisms underlying DC-controlled immune responses in allergic diseases [5].

Benito-Villalvilla et al. demonstrated in 2022 that differentiation of monocytes in the presence of allergoid-mannan conjugates leads to the development of tolerogenic DCs (tolDCs) by epigenetic and metabolic reprogramming in non-atopic as well as allergic subjects [6]. These tolDCs caused the development of forkhead box protein 3 (FOXP3)-positive regulatory T cells and decreased production of the pro-inflammatory cytokine TNF-⍺ and miRNA-155 while increasing the levels of anti-inflammatory miRNA-146a/b [6]. Moreover, they mediated a metabolic shift from Warburg metabolism to OxPhos and therefore represent promising candidates to restore tolerance to allergens in allergen-specific immunotherapy (AIT) [6].

The Krebs cycle intermediate itaconate is produced by aconitase decarboxylase, which is encoded by immune-responsive gene 1 (*Irg1*) and displays immune-modulating properties, as reviewed in 2020 by Li and colleagues [7]. According to Jaiswal et al., the investigation of *Irg1*-deficient DCs revealed an enhanced uptake of allergens and antigen presenting capability to CD4^+^ T cells and therefore increased Th2 effector functions [8•]. Additionally, this enhanced DC activation was associated with increased levels of eosinophilic airway inflammation and production of Th2-cytokines (IL-4 and IL-13) as well as allergen-specific IgE production after the sensitization with HDM [8•]. Thus, itaconate seems to be a key metabolite to suppress mitochondrial oxidative damage and pathogenic inflammation [8•].

Based on a shrimp protein extract-induced BALB/c mouse allergy model, Sun et al. reported distinct differences in the lipid profile and metabolism of spleen DCs of mice being sensitized and challenged with shrimp extract [9]. The authors concluded that lipid metabolism is involved in shrimp allergy and might influence inflammatory immune responses [9]. The supplementation of exogenous lipid glyceryl trioleate affected the immune function of DCs by decreasing the expression of the Th2-associated gene *Il4* and increasing the Th1-related gene *Il12a* and therefore improved the Th1/Th2 immune skew [9]. The C16 lipid ceramide also showed regulatory effects on DC by decreasing the expression of *Stat3* and *Rela,* both being involved in cell signaling pathways that regulate allergic reactions [9]. Therefore, glyceryl trioleate and C16 ceramide might represent potential candidates for dietary supplementation to reduce shrimp allergy [9].

Jaiswal and colleagues showed in 2020 that exposure to the fungal allergen *Alternaria alternata* promotes the accumulation of neutrophils in the airways and changes in pyruvate kinase isoenzyme M2 (PKM2) expression, which is associated with the release of both pro-inflammatory- (IL-6, IL-33, and TNF-⍺) and Th2-cytokines (IL-5 and IL-13) as well as acute airway inflammation [10]. The authors proposed that sensitization with *Alternaria* leads to a cycle of glycolytic reprogramming and PKM2 regulation, accompanied by acute activation of lung APCs [10].

Finally, Svedberg et al. showed alveolar macrophages (AlvMs) to be hyporesponsive to Th2 cytokine IL-4 due to restricted activation of IL-4 receptor after infection with helminths [11•]. Here, in contrast to peritoneal or lung tissue macrophages, AlvMs were characterized by a dysregulated metabolism with impaired glycolysis *in vivo* [11•]. However, when isolated directly from the lung, AlvMs restored their responsiveness to IL-4. Hence, the pulmonary niche might regulate AlvM responsiveness to IL-4, demonstrating that the tissue environment plays a critical role in regulating the metabolic activity and responsiveness of macrophages to Th2 cytokines [11•].

In summary, recent studies confirmed the critical role of APC metabolism in the development and regulation of Th2 responses highlighting the importance of further investigating the underlying mechanisms.

### ILC2s

In the context of allergen-induced airway inflammation, innate lymphoid cells type 2 (ILC2s) were found to display a distinct metabolic phenotype with their effector function mainly relying on FAO [12•]. Here, proliferation and pathologic responses of *ex vivo*-isolated ILC2s were dependent on the uptake and storage of lipids [12•]. Mechanistically, ILC2-metabolism was controlled by mammalian target of rapamycin (mTOR), regulating the expression of diacylglycerol O-acyltransferase 1 (Dgat1) and peroxisome proliferator-activated receptor gamma (PPAR-γ) [12•]. The upregulation of Dgat1-dependent formation of lipid droplets was shown to serve as a protective mechanism of ILC2s against lipotoxicity in order to allow for the accumulation of large amounts of lipids during activation [12]. The metabolic sensor PPAR-γ is also involved in the regulation of Th2 responses and regulates the expression of genes involved in the storage of fatty acids and the metabolism of glucose (reviewed in 2011 by Tyagi et al. [13]). In ILC2s, PPAR-γ regulates fatty acid uptake and thereby proliferation and cytokine production (IL-5 and IL-13) [12•]. Additionally, PPAR-γ depletion was shown to reduce ILC2- and eosinophil-infiltration in the lung *in vivo* [12•]. Furthermore, a ketogenic diet (restricting glucose availability) impairing lipid metabolism resulted in reduced airway infiltration of ILC2s and eosinophils *in vivo* as well as reduced IL-5 and IL-13 production in lung tissue [12•].

Fali et al. also showed PPAR-γ to be highly expressed in ILC2s and being required both for their proliferation and production of the effector cytokines IL-5 and IL-33 by controlling ILC2 energy metabolism (fatty acid uptake) and sensing of IL-33 [14]. In line with this, pharmacological inhibition of PPAR-γ was shown to block ILC2-dependent acute lung inflammation [14].

Apart from the uptake and storage of lipids, the generation of free fatty acids through autophagy was shown to be important to maintain ILC2 homeostasis and effector function [15]. Impaired autophagy, through the deletion of autophagy related 5 (Atg5), resulted in increased apoptosis via reduced nuclear factor kappa-light-chain-enhancer of activated B cells (NF-κB)-signaling and decreased secretion of IL-5, IL-13, IL-9, and IL-6 from *ex vivo*-isolated ILC2s [15]. The loss of autophagy also changed the metabolic phenotype of ILC2s with impaired FAO and inhibition of the Krebs cycle while promoting glycolysis [15].

Metabolic reprogramming in ILC2s is associated with the accumulation of dysfunctional mitochondria and thereby enhanced production of ROS as well as impaired homeostasis and Th2-cytokine production [15]. This metabolic change of ILC2s upon loss of autophagy was found to reduce the development of allergen-induced airway inflammation *in vivo* [15].

Besides FAO, also methionine metabolism as well as overall mitochondrial respiratory capacity regulates cytokine secretion through STAT3 in *ex vivo*-isolated, IL-33-activated ILC2s [16]. Here, the impairment of either the methionine cycle or mitochondrial translocation of STAT3 reduced IL-5- and IL-13-levels in lung tissue and decreased ILC2- and eosinophil-airway infiltration *in vivo* [16].

In addition to STAT3, programmed cell death protein 1 (PD-1) was also shown to regulate methionine metabolism [17•]. Here, PD-1 limited ILC2 proliferation through the inhibition of methionine- and glutamine-metabolism [17•]. Moreover, PD-1 regulated the production of IL-5 and IL-13 as well as the survival of ILC2s upon IL-33 stimulation *ex vivo* [17•]. Helou et al. could further show that a PD-1 agonist effectively decreased ILC2-mediated airway inflammation in humanized mice [17•].

Since ILC2s mediate inflammatory responses to inhaled allergens in the airways via the secretion of pro-inflammatory cytokines like IL-5 and IL-13, Howard et al. reported an ILC2 subset that produces IL-10 in allergen-induced airway inflammation (ILC2_10_) [18•]. IL-10 production in *ex vivo*-isolated ILC2_10_s was mediated via the IL-4 receptor and its downstream target STAT6, leading to the upregulation of the transcription factors cellular musculoaponeurotic fibrosarcoma (cMaf) and B lymphocyte-induced maturation protein-1 (Blimp-1) [18•]. IL-10 secretion functioned on the one hand as an autocrine signal downregulating the production of the inflammatory cytokines IL-5 and IL-13 in ILC2_10_s and on the other hand served as a paracrine signal leading to decreased effector function of ILC2s *in vitro* [18•]. ILC2_10_-function and -generation were additionally shown to be dependent on glycolysis for the production of IL-10, thereby shifting their metabolism from the FAO pathway (which is normally used for pro-inflammatory ILC2 effector function) to glycolysis [18•]. In contrast to the glycolysis-dependence of proinflammatory cytokine secretion in other cell types, these results show that ILC2_10_ exhibit anti-inflammatory cytokine production based on glycolysis.

In summary, the current research indicates that ILC2s mainly require FAO to fulfill their effector function. Nevertheless, also other metabolic pathways like methionine- and glutamate-metabolism were shown to contribute to the development of allergen-induced airway inflammation. Moreover, metabolic reprogramming of ILC2s seems to regulate the development of either a pro- or anti-inflammatory phenotype.

### Mast Cells

Data previously published on the metabolism of mast cells suggested that short-term mast cell activation mainly relies on increased glycolysis while OxPhos also contributes to mast cell metabolism upon longer stimulation (reviewed in [19]).

Paruchuru et al. showed in 2022 that mitochondrial microphthalmia-associated transcription factor (MITF) plays an important role in the activation of mast cells [20]. Studying rat basophil leukemia cells (RBL) and mouse bone marrow-derived mast cells (BMMCs), they demonstrated that MITF affects mast cell degranulation and cytokine secretion (TNF-⍺, granzyme B) [20]. Here, ERK1/2-dependent MITF-activation during mast cell activation increased OxPhos activity in the mitochondria, showing a notable contribution of MITF to overall mast cell metabolism [20].

Moreover, STAT3 is a transcription factor activated by the IL-6 family of cytokines. In addition, recent research has shown that STAT3 can also non-canonically regulate OxPhos by modulating electron transport chain (ETC) activity [21–23]. Erlich et al. recently showed that STAT3-controlled ATP-production via the OxPhos pathway is essential for mast cell exocytosis [24]. In a follow-up study, the authors demonstrated that treatment of mouse and human mast cells with STAT3 inhibitors that specifically target STAT3 in mitochondria significantly reduced mast cell exocytosis and release of TNF-α [25••]. This reduction in mast cell activation was caused by a reduced OxPhos activity and STAT3 serine 727 phosphorylation [25••]. In a mouse model of OVA-induced allergic asthma, STAT3 inhibition significantly reduced eosinophilia and neutrophilic as well as blood histamine levels [25••], suggesting mitochondrial STAT3 to be a promising target to prevent mast cell activation and its associated pathology.

### Eosinophils

In addition to mast cells, eosinophils, with their ability to bind allergen-specific IgE and release a plethora of inflammatory mediators, are highly important effector cells in certain allergic contexts. However, so far, the immune metabolism of eosinophils is under-studied. In 2020, Jones et al. demonstrated that human eosinophils activated by cytokines (IL-3, IL-5, GM-CSF) perform glycolytic metabolism as well as mitochondrial respiration, therefore indicating metabolic plasticity [26•]. The mitochondria might play an important role in eosinophil activation as being demonstrated by the formation of Krebs cycle intermediates from glucose and glutamine [26•]. IL-5-driven eosinophil metabolism was shown to be dependent on the STAT5/PI3K/Akt-signaling pathway, which was confirmed by western blot analysis as well as measurements of extracellular acidification rates (ECAR) and oxygen consumption rates (OCR) [26•]. The authors claim that further studies on eosinophil metabolism could reveal new metabolically targets to treat eosinophil-mediated diseases [26•].

### Th2 Cells

Th2 cells are key drivers of allergic responses by promoting the activation, differentiation, proliferation, and class-switch recombination of B cells producing allergen-specific IgE antibodies. Therefore, understanding the metabolic changes associated with Th2-differentiation and -effector function has the potential to improve allergy treatment.

Stark et al. recently reviewed the metabolic requirements of Th2 cells, finding Th2 differentiation to be correlated with metabolic adaption [27]: As the metabolic requirements are different between naïve T cells and Th2 cells, metabolites and co-factors derived from the respective’s cell metabolism can either influence gene expression, modify chromatin structure, interact with transcription factors, or influence mRNA stability in activated T cells [27]. While naïve, resting T cells in lymph nodes mainly rely on oxidative lipid metabolism, after activation, changes to glycolysis by upregulation of glucose transporter 1 (Glut1), mTOR, and hypoxia inducible factor 1 subunit alpha (HIF-1α) were described [27].

Th2 differentiation is correlated with changes in chromatin structure and therefore, gene accessibility in many gene loci, including open chromatin regions for loci involved in Th2-differentiation and lipid metabolism and the Th2-regulator PPAR-y (see above, [27, 28])*.* In a single-cell RNA analysis involving lung-, lymph node-, and airway-cells from an HDM-mouse model, Tibbit and colleagues found that airway cells belonging to a Th2-related gene cluster (further termed “Th2 cells” for simplicity reasons) had a distinct gene expression pattern and displayed enhanced locus accessibility for genes like *Igfbp7*, *Pparg*, *Il6*, and *Plac8* [28]*.* In addition to genes related to Th2-differentiation, like *Gata3*,* Il13*,* Il5*,* Il4*, and *Pparg*, also *Hif*, *Plac8*, and *Igfbp7* were found to be upregulated by these cells [28]. As *Pparg* encodes for PPAR-y, these results further underline the previously described dependence of Th2 cells on lipid metabolism [28].

As first shown by Tibbitt et al. [28] and later confirmed by Healey et al. [29], metabolically, in vitro-differentiated Th2 cells were shown to display the highest levels of glycolysis of all T helper cell subsets while also having a high mitochondrial oxygen consumption. Furthermore, Th2 cytokine secretion was shown to be glycolysis-dependent, while *ex vivo*-isolated BALF Th2 cells from a HDM-mouse model showed no enrichment of genes related to glycolysis [28]. These contradictory results may be explained by the finding that human CD4^+^ T cells were shown to pre-accumulate untranslated mRNA related to proteins involved in both glycolysis and fatty acid synthesis for direct translation after activation [30]. In contrast, metabolic pathways related to FAO and fatty acid synthesis (FAS) were upregulated and displayed higher gene accessibility (e.g., of the fatty acid transporter *Cpt1a* [27, 28]). Here, inhibition of glycolysis, and to a lower extend either inhibition of fatty acid synthesis( FAS) or FAO, reduced both Th2-differentiation and airway inflammation *in vivo* [28]. The authors concluded that in BALF-derived Th2 cells, lipid metabolism plays a role in Th2-mediated airway inflammation, while glycolysis promotes Th2 cell differentiation [28].

Activated T cells increase their uptake of glutamine and lack of glutamine was shown to impair both their cytokine secretion and proliferation [27]. In 2021, Healey et al. analyzed the bronchoalveolar lavage fluid (BALF) of asthmatic patients and found hints of a generally enhanced metabolism (as shown by higher lactate concentrations after allergen challenge) and markers for both increased glycolysis and glutamine metabolism [29]. In mice sensitized to *Alternaria alternate* extract, enhanced OxPhos, glucose, and glutamine metabolism were observed in both Th2 and Th17 cells, while Th17 cells also had a higher potential for glutamine metabolism [29]. In another mouse model, using HDM + LPS for sensitization and HDM for the challenge, both Th2 cytokine production and cell infiltration were reduced when either Glut1 or the glutamine-metabolizing enzyme glutaminase were inhibited [29]. Moreover, glutaminase inhibition also decreased airway resistance, suggesting that the distinct metabolic profiles of Th2/Th17 cells may allow for new treatment strategies in the future [29].

Another interesting pathway that is usually not in the immunologists’ focus is tryptophan metabolism, including the kynurenine pathway [27, 31]. Here, the rate-limiting enzyme of tryptophan metabolism, [31] indoleamine 2,3-dioxygenase (IDO), was found to both promote Th2 cell function and inhibit Th1 responses, while also circulating IgE levels were shown to be dependent on IDO expression [27]. Even more, 3-hydroxyantranylic acid, quinolinic acid, and to a lower extent 3-hydroxykynurenine (metabolites of the kynurenine pathway and thereby involved in tryptophane catabolism [31]), triggered apoptosis in Th1 cells while having no to little effect on Th2 cell viability [32]. These data indicate that tryptophan metabolism and IDO may play a significant role in Th2 responses.

Differentiation of Th2 cells takes place in germinal centers under hypoxic conditions. Concerning the metabolic requirements of differentiating Th2 cells, Cho et al. found levels of HIF to depend on mTOR in activated CD4^+^ T cells [32]. While hypoxia was not influencing the differentiation of either Th1 or Th2 cells, surprisingly, upon CD4^+^ T cell activation, HIF-1α and HIF-2α were shown to be stabilized not only under hypoxic conditions but also under normoxic conditions (21% pO_2_) [33]. However, HIF-1α-deficient CD4^+^ T cells had a significantly decreased ECAR compared to WT cells after T-cell receptor stimulation under both normoxic and hypoxic conditions, indicating a strong contribution of HIF-1α to glycolysis [33]. As the loss of HIF-1 in CD4^+^ T cells also resulted in decreased secretion of IL-4, IFN-γ, and reduced antigen-specific antibody production, the authors concluded that HIF-1 and glycolysis are directly connected to T cell effector function [33].

Early stages of IL-4-dependent Th2-differentiation and effector function are driven by the transcription factors STAT3, STAT6, GATA3, while later stages depend on PPAR-γ (reviewed in [27]). Interestingly, Th2 cells were described to require tissue-derived cytokine production (IL-25, IL-33, and thymic stomal lymphopoietin (TSLP), e.g., in the lung) to become competent IL-5 and IL-13 producers, while a loss of these signals did not affect the T cell priming in the lymph nodes [34, 35].

Recently, Yagi and colleagues found that inhibition of pyruvate dehydrogenase kinase (PDK) (which catalyzes the generation of acetyl-CoA from glycolysis-derived pyruvate) suppressed the differentiation of IL-5- and IL-4-secreting Th2 cells [36]. In their experimental setting, PDK inhibition suppressed PPAR-y while GATA3 was unaffected, suggesting that PDK inhibition affects only the later stages of Th2-differentiation [36].

Regarding the role of GATA3 in Th2-differentiation, Tiwari and colleagues reported that nuclear receptor subfamily 1 group D member 1 (Nr1d1) suppressed Th2 cell differentiation and attenuated asthma by acting as a transcriptional repressor of the GATA3 promoter [37••]. Furthermore, Nr1d1-deficient T cells had significantly higher levels of IL-4, IL-5, IL-13 (mRNA and protein), and GATA3 (mRNA) compared to WT controls. Interestingly, either the overexpression of Nr1d1 or the treatment with its ligand (SR9011) protected mice against lung inflammation in an asthma model (as shown by lung histology, reduced histological scores, IL-4-, IL-5-, and IL-13-levels, as well as reduced GATA3 expression) [37••]. In a genome-wide ChIP-Seq analysis, the authors were able to identify further interactions of Nr1d1 with the regulation of metabolism and immunity, making Nr1d1 a highly promising target molecule for asthma treatment [37••].

In summary, the metabolism of Th2 cells appears to be both highly glycolytic while also exhibiting a high rate of OxPhos. As activation of both lipid- and glutamine metabolism has also been described in activated Th2 cells, this high rate of OxPhos may be due to mitochondrial utilization of fatty and amino acids. Mechanistically, both enhanced glycolysis and Th2 cytokine secretion were shown to depend on HIF-1, suggesting an intricate connection between Th2 metabolism and effector function. Additionally, inhibition of fatty acid and glutamine metabolism was shown to reduce Th2 inflammation, suggesting the picture to be more complex. In this context, further in-depth analyses are required to better understand the involvement of metabolism in Th2-differentiation and -activation.

## Modulation of Immune Metabolism to Improve the Treatment of Allergies

**Fig. 1:**
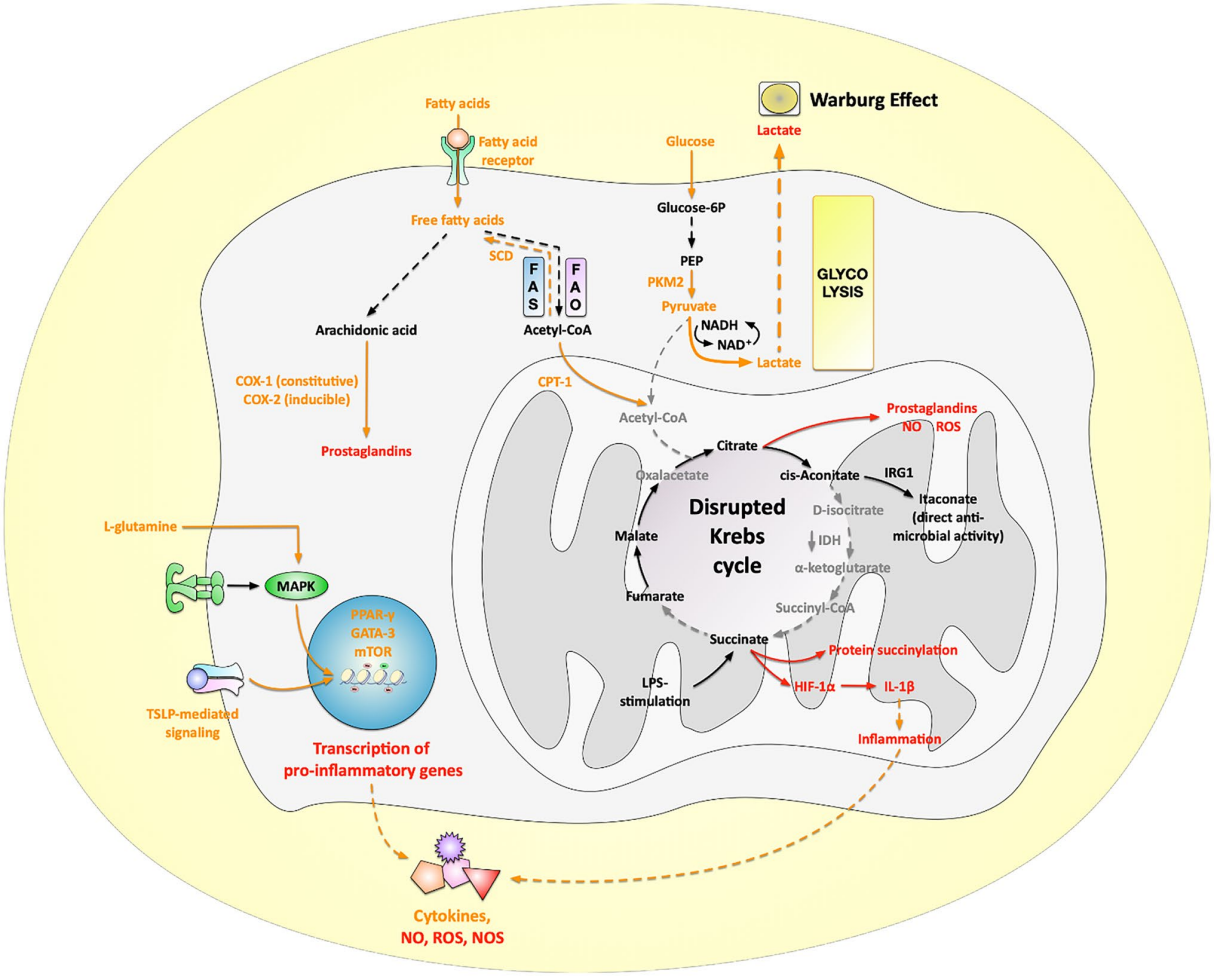
Currently investigated metabolic targets to improve the treatment of allergic diseases. As detailed in this review, cells involved in allergic diseases frequently display metabolic adaptations, e.g., increased glycolytic rates and a disrupted Krebs cycle, changes in fatty acid metabolism leading, for example, to the production of pro-inflammatory prostaglandins, or alterations in amino acid metabolism resulting in altered intracellular signaling events. The presented overview indicates the currently targeted molecules and pathways in orange, while molecules and events contributing to allergic inflammation are indicated in red. For more information see text. Abbreviations: PEP, phosphoenolpyruvate; PKM2, pyruvate kinase isozyme M2; N/ROS, reactive nitrogen/oxygen species; NO, nitric oxide; FAO, fatty acid oxidation; FAS, fatty acid synthesis; SCD, stearoyl-coenzyme A desaturase; CPT-1, carnitine palmitoyltransferase 1; COX-1/2, cyclooxygenase 1/2; IRG1, immune-responsive gene 1; HIF-1α, hypoxia-inducible factor 1α; PPAR-y, peroxisome proliferator-activated receptor gamma; GATA-3, GATA-binding protein 3; mTOR, mammalian target of rapamycin; TSLP, thymic stromal lymphopoietin

It has become clear that altering the metabolic state of immune cells also affects their effector functions. Therefore, targeting metabolic pathways may prove a viable strategy for developing new therapies. In this part, we summarize recent research that has tested drugs that target either (I) amino acid- or (II) fatty acid-metabolism in animal allergy models or in human clinical trials, as well as (III) compounds derived from natural resources that were found to suppress allergic inflammation *in vivo* by altering the respective’s cell metabolism (summarized in Fig. 1).

### Targeting Amino Acid Metabolism

Imbalances in amino acid metabolism can lead to higher ROS- and nitric oxide (NO)-production from airway epithelial cells, impairing lung function in asthmatics [38]. Hu and colleagues showed that in an OVA-induced mouse asthma model, tryptophan metabolites played an essential role in altering the T_reg_/Th17 balance, OVA-specific IgE production, IL-5-, IL-6-, and IL-17A-levels, as well as lung inflammation during AIT [39]. Therefore, some in vivo and clinical studies have tried to target amino acid metabolism to develop new asthma treatments [38, 40•, 41]. For example, Kim and colleagues found that in an OVA-induced mouse asthma model, L-glutamine depletion could impair MAPK phosphatase (MKP)-1 activation, which led to prolonged phosphorylation of p38 MAPK and cytosolic phospholipase A2 (cPLA2), resulting in predominant neutrophilic Th1- instead of eosinophilic Th2 responses in lung tissue (Fig. 1) [40•]. Moreover, a recent patent indicated the inhibition of arginase (an enzyme converting L-arginine to urea and ornithine) to be another target for allergy treatment [41]. This idea was proven in another clinical study performed by Holguin et al. where they showed that 2 weeks of treatment with L-citrulline (a precursor of L-arginine) improved lung function in obese asthmatics [38]. Taken together, these recent reports support the idea that modulating amino acid levels may be a novel strategy to improve asthma treatment.

### Targeting Fatty Acid Metabolism

**Fig. 2:**
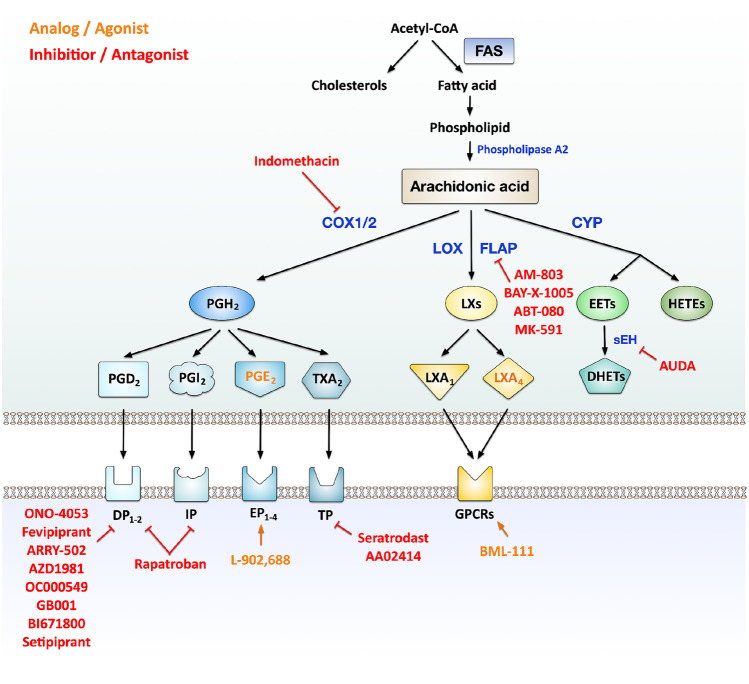
Arachidonic acid metabolism and currently tested targets for the treatments for allergies. Acetyl-CoA can be used to synthesize either cholesterols or fatty acids by fatty acid synthesis. Fatty acids are the major component of phospholipids, and arachidonic acid (AA) is released by phospholipase A2 catalysis. Cyclooxygenases 1/2 (COX-1/2) metabolize AA to prostaglandin H2 (PGH_2_) which is then converted to different prostaglandins (PGD_2_, PGI_2_, PGE_2_, and TXA_2_) that bind to their specific receptors. The enzymes lipoxygenase (LOX) and lipoxygenase-activating protein (FLAP) metabolize AA to lipoxins (LXs), including LXA_1_ or LXA_4_, which are known to bind to G-protein-coupled receptors. Cytochrome P450 metabolizes AA to hydroxyeicosatetraenoic acids (HETEs) or epoxyeicosatrienoic acids (EETs), and EETs are then further metabolized to dihydroxyeicosatrienoic acids (DHETs) by the enzyme soluble epoxide hydrolase (sEH). The currently tested drugs targeting AA metabolic pathways for allergy are also indicated in Fig. 2: the color orange represents treatment with either analogs or receptor agonists, while red color represents treatments with either inhibitors or receptor antagonists. For more information, see text. Abbreviations: FAS, fatty acid synthase; COX1/2, cyclooxygenase 1/2; PGH_2_, prostaglandin H2; PGD_2_, prostaglandin D2; PGI_2_, prostacyclin; TXA_2_, thromboxane A2; DP, prostaglandin D2 receptor; IP, prostacyclin receptor; TP, thromboxane A2 receptor; LOX, lipoxygenase; FLAP, 5-lipoxygenase-activating protein; LXs, lipoxins; GPCRs, G-protein-coupled receptors; CYP, cytochrome P450; HETEs, hydroxyeicosatetraenoic acids; EETs, epoxyeicosatrienoic acids; sEH, soluble epoxide hydrolase; DHETs, dihydroxyeicosatrienoic acids

Within the cell, fatty acids are generated from acetyl-CoA, which is mostly derived from the breakdown of glucose. Fatty acids can be used to synthesize either cholesterols [42], phospholipids, or arachidonic acid (AA) catalyzed by the enzyme phospholipase A2 (Fig. 2) [42].

Disordered lipid metabolism can influence immune cells’ effector functions. Especially both fatty acids and the metabolites derived from AA were reported to regulate pathogenesis in allergic diseases such as asthma [42]. Besides, dietary fatty acids (e.g., short chain fatty acids (SCFAs) like propionate and butyrate, or n-3 polyunsaturated fatty acids (PUFAs) like docosahexaenoic acid (DHA)) have also been demonstrated to influence allergic diseases including asthma, allergic rhinitis, atopic dermatitis, and food allergy [43]. Therefore, several new drugs, either targeting lipid metabolism or supplementing specific dietary fatty acids, have been tested *in vitro*, *in vivo*, and in clinical trials [42, 44–47, 48•, 49•, 50]. Here, we provide a brief summary and classification of recently developed therapeutic approaches targeting either (I) SCFAs or PUFAs or (II) AA metabolites (summarized in Fig. 2).

#### SCFAs or PUFAs

The therapeutic potential of SCFAs in allergy is still controversial. For example, Folkerts et al. found that the SCFAs propionate and butyrate could inhibit both IgE- and non-IgE-mediated degranulation of either peripheral blood mononuclear cell-derived human mast cell or BMMCs *in vitro* [44]. Detailed mechanistic analyses revealed that butyrate treatment suppressed FcεRI-mediated expression of bruton’s tyrosine kinase, spleen tyrosine kinase, and linker for activation of T cell, which were important for mast cell activation [44]. On the contrary, Shi et al. showed that *in vitro* stimulation of basophils purified from healthy donors with either propionate or butyrate could promote IgE-mediated degranulation, IL-13 secretion, and CD69 expression [45]. These results indicated that SCFAs differently affect the cell types involved in allergic responses. Here, further comprehensive *in vivo* studies are needed to better understand the potential of SCFA for the treatment of allergic diseases.

Besides SCFAs, PUFAs were also tested for their potential to improve the treatment of allergic responses. A recent study performed by Son and colleagues tested the therapeutic potential of a free fatty acid receptor 4 (FFA4, a specific PUFA sensor) agonist in an OVA-induced mouse asthma model [46]. They demonstrated that both prophylactic and therapeutic treatment could improve airway inflammation and suppress mucin secretion [46]. Furthermore, the FFA4 agonist also suppressed Th2- (IL-4, IL-5, and IL-13), Th1- (IFN-γ), and Th17- (IL-17A) mRNA expression in the BALF and lung tissue, as well as total IgE levels in serum [46]. Another study by Yu et al. demonstrated that Maresin-2, a lipid mediator biosynthesized from DHA by macrophages, suppressed allergic airway inflammation, Th2 cytokine levels in BALF, and OVA-specific-IgE in serum of OVA-sensitized asthmatic mice [51]. These suppressive effects were found to correlate with the inhibition of the NLRP3 inflammasome and reduced oxidative stress in lung tissue [51].

Taken together, these studies suggest either PUFA administration or targeting PUFA receptors to be promising novel approaches for the treatment of asthma.

#### Arachidonic Acid Metabolites

AA can be metabolized by (I) cyclooxygenase (COX) to generate prostaglandins (PGs), (II) lipoxygenase (LOX) and 5-lipoxygenase-activating protein (FLAP) to generate both leukotrienes and lipoxins (LXs), or (III) cytochrome P450 (CYP) to generate either hydroxyeicosatetraenoic acids (HETEs) or epoxyeicosatrienoic acids (EETs). EETs can be further converted to dihydroxyeicosatrienoic acids (DHETs) by the soluble epoxide hydrolase (sEH) (Fig. 2) [48•, 52].

In the past years, especially COX and PGs have been studied for their immunosuppressive effects and for the application in cancer therapy [53]. Their potential as therapeutic targets for allergy has also been tested recently both *in vivo* and in clinical trials ([42, 47, 48•, 49•, 50]). Van Geffen et al. found that the addition of prostaglandin E2 (PGE_2_) and prostaglandin E2 receptor 4 (EP_4_) agonist L-902,688 (Fig. 2) could enhance the immunosuppressive activity of mouse myeloid-derived suppressor cells (MDSCs) *in vitro* [47]. Furthermore, adoptive transfer of either L-902,688-primed MDSCs or L-902,688 administration alone alleviated lung inflammation in HDM-induced asthmatic mice [47]. Besides, two recent review articles summarized the pharmacologic agents currently tested in human clinical trials for allergic airway diseases, mainly allergic rhinitis and asthma [48•, 49•]. The tested agents could be classified into: (I) COX inhibitors: Indomethacin and Etoricoxib, (II) treatment with PGE_2_ or prostacyclin (PGI_2_) analog OP-41483, or (III) PG receptor antagonists including: thromboxane receptor (TP) antagonists: Seratrodast or AA02414; prostaglandin D2 receptor 1 antagonist: ONO-4053; DP_2_ antagonist: Fevipiprant (QAW039), ARRY-502, AZD1981, OC000549, GB001, BI671800, Setipiprant (ACT-129968); and the dual antagonist for DP_2_ and TP: Ramatroban (Fig. 2) [48•, 49•]. Most of the tested drugs indeed improved allergic symptoms during clinical trials [48•, 49•]. Interestingly, the COX-inhibitor Etoricoxib showed no benefits on bronchial responsiveness from asthmatics, and the PGI_2_ analog OP-41483 did neither improve lung function nor sputum eosinophils numbers in mild asthma patients [48•, 49•].

Besides targeting the COX pathways for developing novel treatments for allergy, current research focusing on the other metabolic pathways metabolizing AA is relatively limited (Fig. 2). FLAP-inhibitors AM-803, BAY-X-1005, ABT-080, and MK-591 as well as LXA4, LXA4-analog 5(S),6(R)-LXA4 methyl ester, and LXA4 receptor agonist BML-111 have been tested in either phase I or phase II clinical trials where they collectively improved lung function in asthmatic patients [42, 48•]. In addition, a recent study by Jiang et al. showed that treatment with the sEH inhibitor AUDA in an OVA-induced mouse asthma model reduced inflammatory cell infiltration, as well as IL-13-, IL-17-, and MMP-9-expression in lung tissues [50]. Taken together, targeting either AA metabolic pathways or its metabolites has shown promising results in both *in vivo* allergic models and human clinical trials.

### Endocannabinoids

Endocannabinoids are endogenously synthesized lipid-derived molecules in the mammalian system [54], which can modulate both immune responses and overall metabolism [54]. Although the role of endocannabinoids in allergy is currently controversial [54], some recent studies demonstrated that either the administration of cannabidiol or the suppression of endocannabinoid degradation by inhibitors could improve asthma in vivo [55, 56]. A review from Angelina et al. recently summarized the findings until 2020 regarding the therapeutic potential of cannabinoids on allergic rhinitis, allergic asthma, atopic dermatitis, allergic contact dermatitis, and food allergy [54]. In brief, the mechanisms by which cannabinoids improve allergic symptoms included (I) regulating cytokine production and (II) suppressing mast cell- and eosinophil-activity [54]. These results demonstrate that further investigation on either ectopic administration or targeting endocannabinoid pathways might be an option for developing new therapies for allergies.

### Other Compounds Derived from Natural Resources

The immune-metabolic therapeutic potential of natural, bioactive compounds for treating different diseases has been investigated in recent years [57]. Studies also demonstrated that natural bioactive compounds could improve allergic symptoms in mouse allergy models by modulating metabolic pathways [58, 59]. Liu and colleagues demonstrated that Pterostilbene, a stilbenoid found in blueberry and vines, could attenuate HDM-induced allergic inflammation by inhibiting both the Glut1/mTOR/GATA3-axis and glycolysis in Th2 cells *in vivo* [58]. In addition, Shou et al. showed that Paeoniflorin, the ingredient in Chinese peony, could suppress airway inflammation and hyper-responsiveness in OVA-induced asthmatic BALB/c mice [59]. Metabolomic and microarray-based RNA analysis revealed the therapeutic effects of paeoniflorin to be based on regulating fatty acid metabolism in lung tissues [59].

## Conclusions

In the last years, we have learned a lot about the complex immune-metabolic phenotype of the distinct cell types contributing to the establishment and maintenance of allergic sensitization. Here, recent reports have both confirmed and analyzed in more detail the importance of glycolysis and fatty acid metabolism for the development and airway inflammation initiated by epithelial cells. Moreover, the initial findings of ILC2s mainly relying on FAO have been substantiated by mechanistic studies showing fatty acid metabolism in ILC2s to strongly depend on PPAR-γ and autophagy. In addition, metabolic pathways like methionine- and glutamate-metabolism were also shown to contribute to the ILC2-dependent development of allergen-induced airway inflammation. Also, first results have been obtained for eosinophils showing a fascinating metabolic plasticity with these cells relying on both glycolytic metabolism as well as mitochondrial respiration. New results on mast cell metabolism have revealed a non-canonical role of STAT6 in the regulation of ETC activity and mast cell degranulation that could be used as a treatment target to suppress mast cell-mediated inflammation. The metabolic adaptations in Th2 cells appear to be complex with these cells being highly glycolytic while also exhibiting a high rate of mitochondrial OxPhos, potentially fed by lipid- and glutamine metabolism. Here, all three pathways were shown to differentially contribute to Th2 effector function and therefore allergic inflammation.

Already early on, immune-metabolic changes in activated immune cells were identified as a promising target to improve disease treatment [60]. The studies published in the last 3 years regarding the development of novel therapeutic agents that target metabolic pathways in allergies have initially demonstrated the potential of many target pathways and molecules to improve allergy treatment. Here, a major focus (with promising results) has been to modulate the production of pro-inflammatory mediators derived from fatty acids. However, so far, these results have not yet found widespread application in human clinical trials. In this context, future drug development may benefit from further studying the detailed mechanisms of immune metabolism during allergic sensitization, allergic reactions, and allergy treatment.

## Abbreviations

AA: Arachidonic acid
AIT: Allergen-specific immunotherapy
AlvM: Alveolar macrophage
Atg5: Autophagy related 5
BALF: Bronchoalveolar lavage fluid
Blimp-1: B lymphocyte-induced maturation protein-1
BMMCs: Bone marrow-derived mast cells
CD[X]: Cluster of differentiation [X]
cMaf: Cellular musculoaponeurotic fibrosarcoma
COX1/2: Cyclooxygenase 1/2
cPLA2: Cytosolic phospholipase A2
CYP: Cytochrome P450
DC: Dendritic cell
Dgat1: Diacylglycerol O-acyltransferase 1
DHA: Docosahexaenoic acid
DHETs: Dihydroxyeicosatrienoic acids
DOX: Doxycycline
ECAR: Extracellular acidification rate
EETs: Epoxyeicosatrienoic acids
EP4: Prostaglandin E2 receptor 4
ETC: Electron transport chain
FAO: Fatty acid oxidation
FAS: Fatty acid synthesis
FFA4: Free fatty acid receptor 4
FLAP: 5-Lipoxygenase-activating protein
FOXP3: Forkhead box protein 3
Glut1: Glucose transporter 1
HDM: House dust mite
HETEs: Hydroxyeicosatetraenoic acids
HIF-1α: Hypoxia inducible factor 1 subunit alpha
IDO: Indoleamine 2,3-dioxygenase
ILC2: Innate lymphoid cells type 2
ILC2_10_: IL-10-producing ILC2s
*Irg1*: Immune responsive gene 1
LOX: Lipoxygenase
LXs: Lipoxins
MDSCs: Myeloid-derived suppressor cells
MITF: Microphtalmia-associated transcription factor
MKP-1: MAPK phosphatase 1
mTOR: Mammalian target of rapamycin
NFκB: Nuclear factor kappa-light-chain-enhancer of activated B cells
NO: Nitric oxide
Nr1d1: Nuclear receptor subfamily 1 group D member 1
OCR: Oxygen consumption rate
OxPhos: Oxidative phosphorylation
PD: Prostaglandin D2 receptor
PD-1: Programmed cell death protein 1
PDK(1): Pyruvate dehydrogenase kinase (1)
PG(E2): Prostaglandin (E2)
PGI2: Prostacyclin
PKM2: Pyruvate kinase isoenzyme M2
PPAR-γ: Peroxisome proliferator-activated receptor gamma
PUFA: Polyunsaturated fatty acid
RBL: Rat basophil leukemia cells
ROS: Reactive oxygen species
SCD1: Stearoyl-coenzyme A desaturase 1
SCFA: Short-chain fatty acid
sEH: Soluble epoxide hydrolase
STAT(-1/3): Signal transducers and activators of transcription 1/3
TP: Thromboxane receptor
TSLP: Thymic stromal lymphopoietin
tolDCs: Tolerogenic DCs
